# Limitations of MALDI-TOF MS in identifying anaerobic bacteremia: challenges in polymicrobial infections and the role of whole-genome sequencing

**DOI:** 10.1128/spectrum.01014-25

**Published:** 2025-06-12

**Authors:** Takuya Hosoda, Masahiro Suzuki, Takahiro Matsuno, Kenjiro Matsui, Koji Ohyama, Yohei Doi

**Affiliations:** 1 Department of Microbiology, Fujita Health University School of Medicine 89305, Toyoake, Japan; 2 Department of Clinical Laboratory, Fujita Health University Okazaki Medical Center 690013, Okazaki, Japan; 3 Department of Clinical Laboratory, Fujita Health University Hospital 97824, Toyoake, Japan; 4 Department of Infectious Diseases, Fujita Health University School of Medicine 89305, Toyoake, Japan; 5 Center for Innovative Antimicrobial Therapy, Division of Infectious Diseases, University of Pittsburgh School of Medicine 12317 , Pittsburgh, Pennsylvania, USA; University of Maryland School of Medicine, Baltimore, Maryland, USA

**Keywords:** anaerobic bacteremia, MALDI-TOF mass spectrometry, whole-genome sequencing, diagnostic microbiology

## Abstract

With the widespread use of matrix-assisted laser desorption ionization time-of-flight mass spectrometry (MALDI-TOF MS), the number of identifiable bacterial species has increased. However, anaerobic bacteremia remains challenging to accurately diagnose due to the diverse range of anaerobic bacteria and the frequent occurrence of polymicrobial infections. Consequently, MALDI-TOF MS often fails to achieve accurate species-level identification in such cases. To address this limitation, we evaluated whole-genome sequencing (WGS) as an alternative method for identifying anaerobic bacteria in blood cultures. Over a 4-year period (April 2020 to March 2024), 69 cases of anaerobic bacteremia were identified, involving 85 bacterial strains. WGS successfully identified 73 strains (89%) at the species level. MALDI-TOF MS accurately identified 43 strains (59%) at the species level and 6 strains (8.2%) at the genus level. Among the 24 discordant strains, 9 species were not included in the database, and 6 species had limited prior reports of bloodstream infections. Additionally, 21 of the 69 cases (30%) were polymicrobial, and WGS revealed 9 cases (13%) in which multiple species had not been identified by MALDI-TOF MS. These results highlight the limitations of MALDI-TOF MS in anaerobic bacterial identification, particularly in polymicrobial infections, and suggest that alternative molecular approaches are necessary to improve diagnostic accuracy.

**IMPORTANCE:**

Accurate identification of anaerobic bacteria remains a significant challenge despite the widespread use of matrix-assisted laser desorption ionization time-of-flight mass spectrometry. While this technology has improved the detection of many bacterial species, some anaerobes remain unidentified due to their absence from reference databases and the difficulties associated with their isolation, particularly in polymicrobial infections. Whole-genome sequencing (WGS) has revealed previously unreported anaerobes and identified polymicrobial infections that were initially misclassified as monomicrobial. Our findings underscore the importance of implementing molecular approaches such as WGS- or PCR-based methods in clinical diagnostics to improve the detection of anaerobic pathogens.

## INTRODUCTION

Anaerobic bacteria are challenging to identify due to their slow growth, strict oxygen requirements, frequent polymicrobial infections, and diverse range of causative pathogens. Matrix-assisted laser desorption ionization time-of-flight mass spectrometry (MALDI-TOF MS) has significantly improved microbial identification in clinical microbiology laboratories, allowing for the rapid and accurate detection of many bacterial species (1, 2). Compared to traditional phenotypic methods, MALDI-TOF MS has enhanced the identification of previously difficult-to-detect bacterial species (3, 4). Consequently, the number of identifiable species has expanded, leading to an improved detection of anaerobic bacteria in clinical specimens (4–6). Despite these advances, the species-level identification rate for anaerobic bacteria using MALDI-TOF MS remains between 65.5% and 90.0% (7–10), underscoring its limitations in distinguishing closely related species and detecting bacteria not included in reference databases. One major limitation is that MALDI-TOF MS reference databases lack many of the anaerobic species that are not frequently isolated from clinical specimens. Anaerobic bacteria constitute a major component of the human microbiota; however, their diversity and clinical significance remain incompletely characterized (11, 12). Although the number of anaerobic species in MALDI-TOF MS databases is increasing (10, 13), species with high isolation frequencies or known pathogenicity are prioritized, while rare or less-characterized species take time to be included. As a result, rare species or those with unclear pathogenicity may be underrepresented in the current databases.

Whole-genome sequencing (WGS) offers a more accurate method for species-level identification of anaerobic bacteria (13, 14). A key advantage of WGS is its ability to identify multiple bacterial species within polymicrobial infections, overcoming the limitations of traditional culture-based methods. WGS can provide a more comprehensive understanding of the species involved in anaerobic bacterial infections.

In this study, we applied WGS to anaerobic bacteria detected from patient blood culture specimens to evaluate its effectiveness in species identification and compare it with MALDI-TOF MS. Our findings highlight the challenges of anaerobic bacteremia identification by MALDI-TOF MS and underscore the need for alternative molecular approaches to improve diagnostic accuracy.

## RESULTS

### Overview of the cases

During the 4-year study period, 85 anaerobic bacterial strains were identified from blood cultures of 69 patients (Table 1). The most frequently isolated genera were *Bacteroides* (including *Parabacteroides* spp. and *Phocaeicola vulgatus*, 35 strains, 41%), *Clostridium* (including *Thomasclavelia ramosa* and *Enterocloster clostridioformis*, 19 strains, 22%), and *Fusobacterium* and *Eggerthella lenta* (5 strains each, 5.9%). Among the 82 strains, 73 strains (89%) were identified to the species level by average nucleotide identity (ANI) analysis, while 9 strains (*Collinsella* spp., *Desulfovibrio* spp., *Finegoldia* spp., *Fusobacterium* spp., *Parvimonas* spp., *Peptoniphilus* spp., *Varibaculum* spp. [one strain each], and *Actinomyces* spp. [two strains]) were classified only to the genus level based on 16S rRNA gene analysis from WGS data. Three strains (3.5%) were identified solely by MALDI-TOF MS due to insufficient DNA extraction from poorly growing cultures.

**TABLE 1:** Classification of bacterial species isolated from blood cultures into pathogens of bacteremia and contaminants

	Number of strains	Identification method
	85	
*Bacteroides* spp.	35	
*Bacteroides fragilis*	16	ANI
*Bacteroides thetaiotaomicron*	5	ANI
*Bacteroides cacae*	3	ANI
*Bacteroides cellulosilyticus*	2	ANI
*Bacteroides hominis*	2	ANI
*Phocaeicola vulgatus*	2	ANI
*Parabacteroides distasonis*	2	ANI
*Parabacteroides goldsteinii*	2	ANI
*Parabacteroides merdae*	1	ANI
*Clostridium* spp.	19	
*Clostridium perfringens*	9	ANI
*Clostridium paraputrificum*	3	ANI
*Clostridium innocuum*	2	ANI/MALDI-TOF MS
*Clostridioides difficile*	1	ANI
*Thomasclavelia ramosa*	2	ANI/MALDI-TOF MS
*Enterocloster clostridioformis*	2	ANI
*Fusobacterium* spp.	5	
*Fusobacterium nucleatum*	2	ANI/MALDI-TOF MS
*Fusobacterium animalis*	1	ANI
*Fusobacterium necrophorum*	1	ANI
*Fusobacterium* spp.	1	16s rRNA
*Eggerthella lenta*	5	ANI
Others	21	
*Actinomyces* spp.	2	16s rRNA
*Desulfovibrio falkowii*	1	ANI
*Desulfovibrio* spp.	1	16s rRNA
*Schaalia turicensis*	2	ANI
*Actinotignum timonense*	1	ANI
*Bifidobacterium longum*	1	ANI
*Christensenella hongkongensis*	1	ANI
*Finegoldia* spp.	1	16s rRNA
*Gordnibacter pamelaeae*	1	ANI
*Mediterraneibacter gnavus*	1	ANI
*Odoribacter splanchnicus*	1	ANI
*Parvimonas micra*	1	ANI
*Parvimonas* spp.	1	16s rRNA
*Prevotella bivia*	1	ANI
*Prevotella intermedia*	1	ANI
*Collinsella* spp.	1	16s rRNA
*Peptoniphilus* spp.	1	16s rRNA
*Prevotella disiens*	1	ANI
*Varibaculum* spp.	1	16s rRNA

### Comparison of MALDI-TOF MS and WGS

MALDI-TOF MS identification results did not fully align with ANI-based WGS analysis (Table 2). Notably, among 73 strains, 43 strains (59%) were identical at the species level, and 6 strains (8.2%) were only identified to the genus level by MALDI-TOF MS, including *Bacteroides thetaiotaomicron, Clostridium perfringens,* and *Fusobacterium nucleatum*. Additionally, *E. clostridioformis* could not be identified, and *Schaalia turicensis* was misidentified as *Actinotignum schaalii*, despite both species being present in the MALDI-TOF MS database. *Bacteroides cellulosilyticus*, *Fusobacterium animalis*, *Mediterraneibacter gnavus*, *Parabacteroides goldsteinii*, and *Parabacteroides merdae* could not be identified by MALDI-TOF MS, as they were not represented in the MALDI-TOF MS database. Among the species not registered, *Bifidobacterium longum* was identified only to the genus level, and *Bacteroides hominis* was identified as *Bacteroides fragilis*.

**TABLE 2:** Discordant identifications between MALDI-TOF MS and WGS

WGS result		MALDI-TOF MS result	Number of strains
Genus-level identification			
	*Bacteroides thetaiotaomicron*	*Bacteroides* spp.	1
	*Clostridium perfringens*	*Clostridium* spp.	1
	*Fusobacterium nucleatum*	*Fusobacterium* spp.	1
Unable to identify or misidentified			
	*Enterocloster clostridioformis*	Not identified	1
	*Parabacteroides distasonis*	Not identified	1
	*Schaalia turicensis*	*Actinobaculum schaalii*	1
Not registered in the MALDI-TOF MS database			
	*Bifidobacterium longum*	*Bifidobacterium* spp.	1
	*Bacteroides hominis*	*Bacteroides fragilis*	2
	*Parabacteroides goldsteinii*	Not identified	2
	*Bacteroides cellulosilyticus*	Not identified	1
	*Mediterraneibacter gnavus*	Not identified	1
	*Parabacteroides merdae*	Not identified	1
	*Fusobacterium animalis*	Not identified	1

### Identification of multiple species through WGS

Polymicrobial bacteremia was observed in 21 of 69 cases (30%) (Table 3). Among them, nine cases (13%) contained multiple anaerobic species that were not successfully identified by MALDI-TOF MS (Table 4). In two cases, only one bacterial species was identified, while in seven cases, no identification results were obtained. WGS analysis revealed that these samples contained multiple species. Several detected species, including *Actinotignum timonense*, *Christensenella hongkongensis*, *Desulfovibrio falkowii*, and *Gordonibacter pamelaeae*, were absent from the MALDI-TOF MS database, contributing to misidentification or failure to identify these strains.

**TABLE 3:** Clinical characteristics and focus of infection in patients with polymicrobial bacteremia

	Age	Sex	Entry	Diseases	Species
1	60	M	Intra-abdominal	Sigmoid colon cancer	*Bacteroides thetaiotaomicron*
					*Eggerthella lenta*
2	83	M	Intra-abdominal	Colon cancer	*Clostridium perfringens*
					*Prevotella intermedia*
					*Bacillus* spp.
3	77	M	Genitourinary	Bladder cancer	*Gordnibacter pamelaeae*
					*Eggerthella lenta*
					*Actinotignum timonense*
					*Schaalia turicensis*
4	48	M	Intra-abdominal	Rectal cancer	*Bacteroides caccae*
					*Bacteroides fragilis*
					*Fusobacterium animalis*
5	69	M	Intra-abdominal	Colon cancer	*Christensenella hongkongensis*
					*Eggerthella lenta*
6	63	M	Intra-abdominal	Intra-abdominal abscess	*Desulfovibrio falkowii*
					*Eggerthella lenta*
7	78	F	Intra-abdominal	Intestinal perforation	*Bacteroides cellulosilyticus*
					*Eggerthella lenta*
8	68	F	Intra-abdominal	Intestinal perforation	*Bifidobacterium longum*
					*Phocaeicola vulgatus*
9	82	F	Intra-abdominal	Intestinal perforation	*Clostridium innocuum*
					*Fusobacterium* spp.
10	74	M	Intra-abdominal	Intestinal perforation	*Clostridium innocuum*
					*Parabacteroides goldsteinii*
11	80	F	Skin and soft tissue	Pressure ulcers and osteomyelitis	*Thomasclavelia ramosua*
					*Schaalia turicensis*
12	73	M	Intra-abdominal	Intestinal ischemia	*Fusobacterium nucleatum*
					*Phocaeicola vulgatus*
13	83	M	Intra-abdominal	Intestinal perforation	*Bacteroides cellulosilyticus*
					*Escherichia coli*
14	86	M	Intra-abdominal	Peritonitis	*Bacteroides fragilis*
					*Klebsiella pneumoniae*
15	89	F	Intra-abdominal	Colon cancer	*Bacteroides fragilis*
					*Streptococcus mitis*
16	79	M	Genitourinary	Fournier gangrene	*Bacteroides fragilis*
					*Streptococcus constellatus*
17	79	F	Intra-abdominal	Acute cholangitis	*Bacteroides fragilis*
					*Klebsiella pneumoniae*
18	70	F	Genitourinary	Stone pyelonephritis	*Bacteroides fragilis*
					*Enterococcus faecalis*
19	83	F	Intra-abdominal	Intestinal perforation	*Bacteroides hominis*
					*Streptococcus anginosus*
20	85	M	Intra-abdominal	Acute cholangitis	*Clostridium perfringens*
					*Klebsiella pneumoniae*
21	83	F	Unknown	Pneumonia	*Enterocloster clostridioformis*
					*Parabacteroides distasonis*

**TABLE 4:** Discordant identifications between MALDI-TOF MS and WGS with multiple bacterial species within the same specimen

	MALDI-TOF MS result	WGS result	
1	Not identified	*Actinotignum timonense* (not registered in database)	*Schaalia turicensis*
2	Not identified	*Bacteroides caccae*	*Bacteroides fragilis*
3	Not identified	*Bacteroides cellulosilyticus* (not registered in database)	*Eggerthella lenta*
4	Not identified	*Christensenella hongkongensis* (not registered in database)	*Eggerthella lenta*
5	Not identified	*Desulfovibrio falkowii* (not registered in database)	*Eggerthella lenta*
6	Not identified	*Gordnibacter pamelaeae* (not registered in database)	*Eggerthella lenta*
7	Not identified	*Enterocloster clostridioformis*	*Parabacteroides distasonis*
8	*Bacteroides thetaiotaomicron*	*Bacteroides thetaiotaomicron*	*Eggerthella lenta*
9	*Clostridium perfringens*	*Clostridium perfringens*	*Prevotella intermedia*

## DISCUSSION

The predominant anaerobic bacteria identified from blood cultures in this study were consistent with previous reports (Table 5), with *Bacteroides* spp. (41%) and *Clostridium* spp. (22%) being the most frequently detected. Importantly, polymicrobial bacteremia was observed in 30% of cases, underscoring the complexity of anaerobic bloodstream infections. Some of these cases presented challenges in isolating pure cultures, making species identification by MALDI-TOF MS difficult. Additionally, some rarely detected or previously unreported species were identified. These findings highlight the limitations of MALDI-TOF MS in accurately detecting less commonly reported anaerobic bacteria, which may contribute to diagnostic delays and misidentifications in clinical laboratories.

**TABLE 5:** Summary of articles published in the literature reporting information regarding the incidence of anaerobic bacteremia

Author	Zahar et al. (15)	Blairon et al. (16)	Vena et al. (17)	Kim et al. (18)	Zouggari et al. (19)	Mortensen et al. (20)	Nagaoka et al. (21)	This study
Year/country	2005/France	2006/Belgium	2015/Spain	2016/Korea	2022/Belgium	2024/Denmark	2024/Japan	2025/Japan
Number of samples	45	39	113	70	105	1,750	520	69
Age	56	61.2	63.4	61	66.4	71	75	76.3
Male sex (%)	27 (60)	24 (61.2)	66 (58.4)	38 (54.3)	64 (61)	926 (53)	295 (56.7)	38 (55)
Isolated bacteria								
*Bacteroides* spp.	60%	63.4%	42.1%	45.7%	32.3%	45%	48%	41%
*Clostridium* spp.	22.2%	12.2%	13.7%	12.9%	15.2%	20%	31%	22%
*Fusobacterium* spp.	2.2%	4.9%	8.4%	1.4%	17.1%	6%	4%	5.9%
Unidentified	–^*a*^	–	–	–	–	4%	4%	
Identification method	Phenotypic method	Phenotypic method	Phenotypic method	Phenotypic method and MALDI-TOF MS	MALDI-TOF MS	Phenotypic method and MALDI-TOF MS	Phenotypic method and MALDI-TOF MS	WGS

^*a*^ –, indicates that the source paper does not include any data.

The main reason for the difficulty in identifying anaerobic bacteria using MALDI-TOF MS is the presence of rarely reported species. Although this study included only 69 cases, 11 species were not included in the VITEK MS database, and another 7 species have rarely been detected (21–25). Expanding the MALDI-TOF MS database to include these species could improve the accuracy of reporting anaerobic bacteremia.

Furthermore, some WGS results of the strains did not match any known species, necessitating the use of 16S rRNA sequences to estimate the genera. It is possible that some of them are previously unknown. Although the clinical significance of some of these species remains uncertain, their absence from reference databases hampers accurate identification. The lack of accurate species information in anaerobic bacterial infections may also limit epidemiological investigations. To improve the accuracy of MALDI-TOF MS identification, it is necessary to accumulate data on species causing anaerobic bacterial infections through WGS analysis.

However, some challenges may still persist even with database improvements. Certain anaerobic bacteria are difficult to isolate from other anaerobic species. For example, *E. lenta* could not be isolated using traditional methods but was detected alongside other species through *in silico* analysis of WGS results. This challenge may arise due to significant differences in growth rates among species and the presence of bacteria that produce extremely small colonies, which remain undetectable even after prolonged incubation. Additionally, some species may preferentially grow in association with other anaerobic bacteria. When multiple species coexist, isolating a pure culture of specific anaerobic bacteria becomes challenging. MALDI-TOF MS is inherently limited in its ability to distinguish multiple bacterial species within the same specimen, reducing its effectiveness in diagnosing polymicrobial anaerobic infections.

MALDI-TOF MS is a valuable clinical tool due to its speed and minimal sample requirements compared to WGS. However, WGS plays a crucial complementary role by enabling the identification of organisms that MALDI-TOF MS cannot reliably detect. Furthermore, incorporating WGS-based findings into reference databases will progressively enhance the diagnostic accuracy of MALDI-TOF MS, particularly for rare anaerobic pathogens and polymicrobial infections.

To address the challenges associated with performing WGS, the development of gene-based direct detection methods, such as PCR, which do not require subculturing, may offer significant clinical advantages. In particular, multiplex PCR targeting anaerobic species commonly associated with bloodstream infections appears to be a promising approach. These methods can enable rapid and accurate identification even in polymicrobial samples, making them potentially more suitable for the diagnosis of anaerobic bacterial infections. Although the broad taxonomic diversity of anaerobic bacteria complicates the creation of comprehensive PCR detection systems, assays focusing on frequently encountered species could still be valuable for both clinical and epidemiological surveillance.

One limitation of this study is that frozen stock samples were used, and some strains exhibited poor growth or failed to revive, potentially leading to an underestimation of diversity. This limitation could have affected the detected species and their proportions. Also, specimens from one hospital were included; therefore, the epidemiology described here may not be generalizable.

In conclusion, the findings underscore the limitations of MALDI-TOF MS in anaerobic bacterial identification and emphasize the need for expanded reference databases or molecular detection methods to improve clinical diagnostics.

## MATERIALS AND METHODS

### Setting

A retrospective observational study was conducted at the Fujita Health University Hospital (1,376 beds) and the Fujita Health University Okazaki Medical Center (400 beds) in Aichi Prefecture, Japan.

### Patients and definitions

Patients with anaerobic bacterial species identified in blood cultures between April 2020 and March 2024 were included in this study. Patients with the same anaerobic bacterial species detected from two sets of blood cultures collected on the same day were considered to have a bloodstream infection. Polymicrobial bacteremia was defined as the presence of two or more bacterial species in a single blood culture bottle or separate culture bottles from the same blood draw. Comorbidities were identified based on the description given by the treating physician in the medical records. An infection focus was defined as the presence of at least two of the following criteria: (i) isolation of bacteria from a focal culture, (ii) radiological or clinical signs of focal infection, and (iii) symptoms of focal infection (21). Mortality was attributed to bacteremia when patients died within 30 days of collection of the positive blood culture.

The median patient age with bacteremia was 76.3 years (SD, 13.9), and 55% were male. The most common comorbidity was malignancy (28 patients, 41%), and the most frequent source of bacteremia was intra-abdominal infection (35 patients, 51%). The 30-day mortality rate was 8.7% (6 patients).

### Blood culture

Blood cultures were processed using the BD BACTEC FX automated system (Becton Dickinson, Sparks, MD, USA). A total of 10 mL of blood was collected per culture bottle and inoculated into anaerobic culture bottles (BACTEC-Plus and -Lytic). The cultures were incubated at 35°C–37°C for up to 7 days. Next, aliquots from positive anaerobic bottles were plated onto the Brucella HK (RS) agar (Kyokuto Pharmaceutical Industrial, Tokyo, Japan) and incubated for more than 48 hours under an anaerobic atmosphere.

Identification of anaerobic bacteria was carried out using VITEK MS (BioMérieux, Métropole de Lyon, France). The captured spectra were analyzed via VITEK MS control and Myla software using *in vitro* database (IVD) version 3.0.

MALDI-TOF MS analysis was performed using bacterial strains isolated from positive blood culture samples obtained during routine diagnostic procedures. Whole-genome sequencing was conducted in batches using cryopreserved strains that had been stored at −80°C in skim milk. Prior to DNA extraction, these strains were cultured under anaerobic conditions on Brucella HK agar.

DNA extraction was performed using the Gentra Puregene Yeast/Bact kit (Qiagen, Venlo, Netherlands) in combination with EZ Beads (AMR, Gifu, Japan). To enhance DNA yield, the protocol was modified by incorporating heat treatment at 95°C for 10 minutes, followed by mechanical cell wall disruption using zirconia beads via vortexing for 5 minutes. Extracted DNA with a concentration of 1 ng/µL or more was selected for WGS. VITEK MS results were used for some strains where adequate DNA could not be collected due to poor growth of preserved strains. These were excluded from the comparison of concordance with MALDI-TOF MS.

WGS was performed using the NextSeq2000 (Illumina, San Diego, CA, USA) with paired-end sequencing (2 × 150 bp), and *de novo* assembly was conducted using SPAdes version 3.13.1.18. The genome data obtained from NGS were analyzed for size, and for assemblies suspected to be from multiple bacteria, separation was performed based on contig size, followed by searches of each assembly.

The whole genome similarity was analyzed by calculating digital DNA-DNA (dDDH) values (22). Genomic data obtained by NGS were registered in the Type Strain Genome Server (https://tygs.dsmz.de) and analyzed pairwise by dDDH with strain type. Strains with dDDH values more than 70% to each other were considered as possibly homologous and analyzed by the ANI method (23). The ANI analysis was performed with OrthoANI (https://help.ezbiocloud.net/orthoani-genomic-similarity/). The ANI values of the genomes obtained by NGS were compared with those of bacterial species downloaded from the NCBI that were of the same species, and those that matched by more than 95% were identified as the same species.

For strains that could not be identified by the dDDH and ANI analysis, BLAST searches for 16S rRNA were performed to determine the genus (24–26).

## Data Availability

Sequence reads of the strains are available under the BioProject accession number PRJNA1244920.
